# The *Phytophthora* RXLR Effector Avrblb2 Modulates Plant Immunity by Interfering With Ca^2+^ Signaling Pathway

**DOI:** 10.3389/fpls.2019.00374

**Published:** 2019-03-28

**Authors:** Zunaira Afzal Naveed, Shaheen Bibi, Gul Shad Ali

**Affiliations:** Mid-Florida Research and Education Center, Department of Plant Pathology, University of Florida, Apopka, FL, United States

**Keywords:** RXLR effector, AVRblb2 effector, Rpi-blb2, calmodulin (CAM), calcium signaling, plant-*Phytophthora* interactions

## Abstract

In plants, subcellular fluctuations in Ca^2+^ ion concentration are among the earliest responses to biotic and abiotic stresses. Calmodulin, which is a ubiquitous Ca^2+^ ion sensor in eukaryotes, plays a major role in translating these Ca^2+^ signatures to cellular responses by interacting with numerous proteins located in plasma membranes, cytoplasm, organelles and nuclei. In this report, we show that one of the *Phytophthora* RXLR effector, Avrblb2, interacts with calmodulin at the plasma membrane of the plant cells. Using deletion and single amino acid mutagenesis, we found that calmodulin binds to the effector domain of Avrblb2. In addition, we show that most known homologs of Avrblb2 in three different *Phytophthora* species interact with different isoforms of calmodulin. Type of amino acids at position 69 in Avrblb2, which determines Rbi-blb2 resistance protein-mediated defense responses, is not involved in the Avrblb2-calmodulin interaction. Using *in planta* functional analyses, we show that calmodulin binding to Avrblb2 is required for its recognition by Rpi-blb2 to incite hypersensitive response. These findings suggest that Avrblb2 by interacting with calmodulin interfere with plant defense associated Ca^2+^ signaling in plants.

## Introduction

*Phytophthora* spp. carry hundreds of effector proteins that are secreted either to the apoplast or inside of host cells (Haas et al., 2009; Raffaele et al., 2010). Most of the translocated *Phytophthora* effectors contain an N-terminal RXLR motif (Arg, any amino acid, Leu, Arg), which is reported to be involved in translocating effectors into host cells and a C-terminal domain with effector activity. RXLR effectors enable Oomycetes to suppress basal immunity (Birch et al., 2008; Brasier, 2009; Fan et al., 2011; Bozkurt et al., 2012) as well as function as avirulence (*Avr*) factors in activating their cognate resistance genes (*R* genes) in the hosts (Anderson et al., 2015). Since the discovery of these RXLR effectors, great efforts are devoted to understanding the molecular mechanisms of how these effectors suppress or trigger plant immunity (Anderson et al., 2015). The RXLR effectors share little sequence homology to characterized proteins thus precluding homology-based functional inferences. Identifying host targets of these effectors is a widely used method for providing clues about their function.

Current findings suggest that RXLR effectors primarily suppress host defense mechanisms (Brasier, 2009; Fan et al., 2011) but also alter other cellular processes (Birch et al., 2008; Bozkurt et al., 2012). Functional annotation as well as *in planta* functional analyses have shown that these effectors affect autophagy (Dagdas et al., 2016), protein degradation and stability (CMPG1) (Bos et al., 2010; Gilroy et al., 2011), kinase and phosphatase signaling (MAPKKK, PP1c) (King et al., 2014; Boevink et al., 2016a,b), transcription (NAC) (McLellan et al., 2013), RNA binding and small RNA biogenesis (Qiao et al., 2013; Du et al., 2015; Jing et al., 2016), protein secretion (Du et al., 2015), endoplasm reticulum stress-mediated immunity (Jing et al., 2016) and brassinosteroid hormone signaling (Saunders et al., 2012). A plant phosphatase BSL1 interacts simultaneously with the PiAvr2 effector and the host disease resistance protein R2 to mediate resistance against *P. infestans* strains that carry avirulent forms of Avr2 (Saunders et al., 2012). Interestingly, although both virulent and avirulent alleles of PiAvr2 interact with BSL1, only the avirulent variants mediate interaction of R2 with BSL1. The PiAvr3a interact with and stabilize the U-box E3 ligase CMPG1 (Bos et al., 2010), which functions in protein degradation and is required for cell death induced by INF1 and some R proteins (Bos et al., 2006; Gilroy et al., 2011). Another RXLR effector, PexRD2 binds to MAPKKKε to interrupt associated signaling pathways involved in defense response (King et al., 2014). Some RXLR effectors interact with susceptibility (S) factors of the host to promote virulence (Boevink et al., 2016b). For example, Pi04089 interacts with a RNA-binding protein KRBP1 to enhance *P. infestans* colonization (Du et al., 2015). Host vesicle-trafficking and secretion mechanisms have also found to be modulated by RXLR effectors. AVR1 that is recognized by R1 to induce HR in the host, is reported to interact and stabilize an exocyst component Sec5 resulting in enhanced defense against *P. infestans* (Du et al., 2015). PiAvr-blb2, another *P. infestans* RXLR effector, interacts with a plant immune protease C14 and prevents its secretion in the apoplast, apparently to prevent degradation of the *Phytophthora* virulence proteins (Bozkurt et al., 2011). *Rpi-blb2*, a coiled-coil-nucleotide-binding leucine-rich repeat (CC-NBS-LRR) receptor type *R* gene has been shown to recognize PiAvrblb2 and trigger hypersensitive response (HR) (Oh et al., 2009). Transformation of an *Rpi-blb2* genomic clone from *S. bulbocastanum*, spanning the entire *Rpi-blb2* gene cassette consisting of its native promoter, coding regions and 3′ untranslated regulatory regions, into potato conferred broad spectrum late blight resistance (Song et al., 2003). In addition, *N. benthamiana* transformed with the same *Rpi-blb2* gene cassette, responded to transient expression of the PiAvr-blb2 by displaying strong HR through SGT1 mediated pathways (Oh et al., 2009). Cross talk between SGT1 and calcium signaling pathways (Nowotny et al., 2003) has been reported to regulate immune responses (Liu et al., 2016).

It is well established that spatio-temporal oscillations in plant cellular Ca^2+^ levels are early events in response to microbes including *Phytophthora* (Tavernier et al., 1995; Zimmermann et al., 1997; Xu and Heath, 1998; Blume et al., 2000; Grant et al., 2000; Lecourieux et al., 2002; Gust et al., 2007; Ranf et al., 2008). Pathogen-induced Ca^2+^-signatures are decoded by various Ca^2+^-binding proteins, among which calmodulin is relatively well studied in numerous plant microbe interactions (Harding et al., 1997; Do Heo et al., 1999; Aharon et al., 2003; Chiasson et al., 2005; Takabatake et al., 2007; Gao et al., 2010; Reddy et al., 2011; Truman et al., 2013; Du et al., 2015). Calmodulin has four Ca^2+^-binding EF-hand motifs. Upon binding Ca^2+^, it undergoes a conformational change, which allows it to bind and modulate the activity of numerous proteins involved in diverse cellular processes including plant defense (reviewd in Kudla et al., 2010; Reddy et al., 2011). Numerous studies have demonstrated involvement of calmodulins and calmodulin-binding proteins including transcription factors, kinases, phosphatases, channels and pumps, and many uncharacterized proteins in plant defense (Reddy et al., 2000; Kim et al., 2002; Aharon et al., 2003; Ali et al., 2003; Choi et al., 2009; Reddy et al., 2011; Poovaiah et al., 2013). Recently, it was reported that the *Pseudomonas syringae* effector HopE1 utilize CaM as a cofactor to target MAP65, which is an important component of cell microtubule network, and reduces the secretion of an immunity related protein PR-1 leading to inhibiting cell wall associated extracellular PTI responses (Guo et al., 2016).

Here we report the interaction of different Avrblb2 homologs with calmodulins (CaMs). We show that this Avrblb2-CaM interaction is independent of amino acid polymorphism at position 69, which is important for Avrblb2 recognition by Rpi-blb2. Using *in planta* functional analyses, we show that the interaction of Avrblb2 to calmodulin is essential for the Avrblb2/Rpi-blb2 effector/R gene-mediated HR response.

## Materials and Methods

### Yeast Two Hybrid Analysis

Yeast Two Hybrid screens were performed according to protocols outlined in the ProQuest^TM^ manual (Invitrogen). The Avrblb2 effectors genes were PCR-amplified using genomic DNA of *P. infestans, P. parasitica* and *P. sojae* using primers listed in Supplementary Table S2. The PCR products were cloned in the Gateway^TM^ pENTR/D-TOPO^®^ entry vector and verified by DNA sequencing. The PiAvrblb2 gene (PITG04090) without its signal peptide was cloned as Gal4-DNA-Binding-Domain (DBD) fusion in the bait pDEST32 vector using the LR clonase^TM^ II kit (invitrogen) for Y2H screens. This construct was transformed into MaV203 yeast cells using the PEG/LiAC method Invitrogen (2012)[ProQuest^TM^ manual www.invitrogen.com]

**FIGURE 1:**
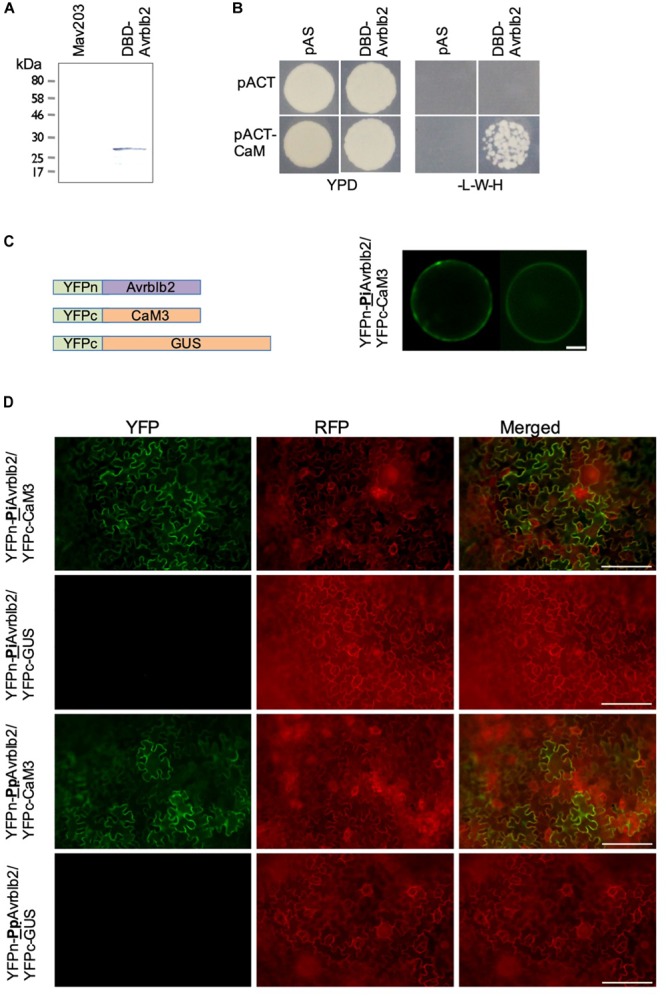
The *Phytophthora* Avrblb2 effectors interact with calmodulin. **(A)** The GAL4-DBD-Avrblb2 fusion protein was expressed in the yeast Mav203 cells. Control, Mav203 cells without any vector [left lane] and Mav203 carrying pDEST32-DBD-Avrblb2 (Avrblb2 fused to the Gal4-DNA binding domain) [right lane] were subjected to Western blot using anti-GAL4-DBD-HRP antibody. **(B)** DBD-Avrblb2 interacted with calmodulin fused to the Gal4-Activation Domain (pACT-CaM) in yeast, as is shown by growth on –L-W-H+3AT plates. pAS and pACT are empty vector, that express the GAL4-DBD and GAL4-ACT domain, respectively, and were used as negative controls. **(C)** Left panel: Schematics of constructs used for BiFC analyses. Avrblb2 was fused to YFPn (YFPn-PiAvrblb2) and CaM3 was fused to YFPc (YFPc-CaM3). GUS fused to YFPc was used as negative control. Right panel: BiFC analyses of the interaction of YFPn-PiAvrblb2 co-expressed with calmodulin YFPc-CaM3 in Arabidopsis protoplasts. Scale bar represents 10 μm. **(D)** *In vivo* BiFC analyses showing interaction of CaM3 with the *Phytophthora infestans* and the *P. parasitica* Avrblb2 homologs at the plasma membrane of leaf cells of *N. benthamiana.* Scale bar represents 100 μm.

Cells expressing Gal4DBD-Avrblb2 fusion protein were selected on -Leu plates. Using an anti-Gal4-DBD-HRP antibody, correct sizes of all Gal4DBD-Avrblb2 fusion was verified using immunoblot analysis (Figure 1A). MaV203 cells carrying pDEST32-Avrlbl2 were transformed with 30 μg of a tomato bait cDNA library in the pDEST22 using the PEG/LiAC method. The Y2H cDNA library from tomato was made using the ProQuest^TM^ Two-Hybrid System in the Gateway^TM^ pDONR222 entry vector^1^. It has a total of 1.04 × 10^7^ primary clones and an average > 1 Kb cDNA size. This library was transferred to the Y2H prey library destination vector (pDEST22) using the LR Clonase II^TM^ *in vitro* recombination kit (Invitrogen). MaV203 cells carrying pDEST32-Avrlbl2 and transformed with the Y2H library were plated on SC-Leu-Trp-His + 25 mM 3AT plates and positive colonies identified. DNA from several independent clones were sequenced, and putative positive clones were retransformed with pDEST32-Avrblb2 into MaV203 for verification. Three additional rounds of Y2H screens were performed.

### *In vitro* CaM-Binding Assays

For *in vitro* CaM-binding assays, all WT and mutant Avrblb2 constructs were expressed as GST fusions in the pDEST15 vector and all calmodulin constructs were expressed as 6X His fusions in pET32GW in *E. coli* BL21 (DE3) pLys S cells. Protein expression, isolation, *in vitro* Co-immunoprecipitation assays and CaM-HRP overlay assays were essentially performed as previously described (Reddy and Reddy, 2002). Briefly, Avrblb2 or calmodulins fusion proteins were induced in 50 mL *E. coli* BL21 (DE3) pLys S cells using 1 mM IPTG for 3 h at 30°C. Cells were pelleted and lysed in 5 mL cell lysis buffer (TBST:50 mM Tris–HCl pH 7.5, 150 mM NaCl, 0.1% Tween-20) containing 200 μg/mL lysozyme and EDTA-free complete protease inhibitors (Cat# 04693132001, Roche) on ice for 30 min. Lysate was centrifuged at 10,000 × *g* at 4°C for 15 min and the supernatant was filtered through 0.22 μm filters (Millipore). Protein concentrations were determined using the Bio-Rad Bradford protein assay, and were adjusted to approximately 1 mg/mL for each sample.

Co-immunoprecipitation assays were done using the Glutathion MagBeads (L00327, GenScript) according to the manufacturer’s protocol with modifications as follows. One hundred μL of Glutathione MagBeads were washed 3 × with TBST. One mL of GST-tagged Avrblb2 fusion protein extracts were added Glutathione MagBeads and incubated at 4°C for 2 h with gentle rotation. Beads were centrifuged at 200 × *g* for 2 min and washed five times with 1 ml TBST. 1 ml of HIS-CaM extracts in TBST containing either 2 mM CaCl_2_ or 5 mM EGTA were added to the GST-Avrblb2-bound Glutathione beads and incubated at 4°C for 2 h with gentle rotation. Beads were washed five times with 1 ml TBST containing either 2 mM CaCl_2_ or 5 mM EGTA. After the final wash, beads were resuspended and boiled in 100 μL SDS sample buffer (60 mM Tris–HCl pH 6.8, 2%SDS, 5% 2-mercaptoethanol, 10% glycerol, 0.005% bromophenol blue). Twenty μl were loaded per lane on 12% PAG in duplicate and run at 150 volts for 1 h in running buffer (25 mM Tris, 192 mM glycine and 0.1% SDS). Proteins were transferred to PVDF membranes in trans-blot buffer (25 mM Tris, 192 mM glycine, 20% methanol) at 100 volts for 1 h. One membrane was probed with anti-GST-HRP antibody (A00866, GenScript; 1:5000 dilution in TBST) and the other was probed with Anti-HIS-HRP antibody (#631210, clontech; 1:10,000 dilution in TBST) according to the manufacturer’s protocols.

For CaM-HRP overlay assays, different Avrblb2 proteins tagged with GST were run on duplicate 12% SDS-PAG and transblotted to PVDF membranes as described above. One membrane was probed with anti-GST-HRP antibody (A00866, GenScript; 1:5000 dilution in TBST) and the other two blots were used in CaM-HRP assays using an AtCaM2-HRP and a previously described standard protocol (Reddy and Reddy, 2002).

### Deletion Constructs and Site-Directed Mutagenesis

Deletion constructs and site-directed mutagenesis was performed using appropriate primers (Supplementary Table S1) using the In-Fusion^®^ HD^®^ Cloning Kit (cat# 638909, Clontech) according to the recommended instructions. All mutants were verified by DNA sequencing.

### BiFC Analysis

Using sequence-verified calmodulin and wild type and mutant Avrblb2 constructs cloned in pENTR/D-TOPO^®^ entry vector were used for making C-terminal YFPc-CaM3 and N-terminal YFPn-Avrblb2 constructs in plant binary BiFC vectors pGSA002-YFPc and pGSA002-YFPn, respectively. These constructs were transformed into *Agrobacterium tumefaciens* GV3101, and each pair of BiFC constructs were transiently expressed in *N. benthamiana* using the *Agrobacterium*-mediated transient transformation system. Confocal images were taken using the Olympus Spinning Disk (Ix81) confocal microscope.

### Hypersensitive Response Analysis in Plants

HR response was analyzed in wild type *N. benthamiana* (Nb/WT) plants or *N. benthamiana* that were stably transformed with the *Rpi-Blb2* gene (Nb/Rpi-blb2) using the *Agrobacterium*-mediated transient expression of Avrblb2 proteins. *A. tumefaciens* GV3101 carrying appropriate WT Avrblb2 or non-calmodulin-binding Avrblb2 constructs were syringe-infiltrated on at least 20 spots into fully expanded leaves of Nb/WT or Nb/Rpi-blb2, and visual HR (cell death) was recorded 2 and 15 days after inoculation. Each experiment was repeated three times.

## Results

### The *Phytophthora infestans* Avrblb2 Interacts With Calmodulins in Y2H Assays and in Plant Cells

To identify putative host targets of the *P. infestans* effector Avrblb2, extensive yeast two hybrid (Y2H) based screenings using mature PiAvrblb2 protein (aa 21–100) as a bait against a *Solanum lycopersicum* cDNA library were conducted. Expression of Gal4-DBD-Avrblb2 fusion protein in MAV203 cells was confirmed by immunoblot analyses (Figure 1A). Multiple Y2H screening resulted in the identification of several putative Avrblb2 targets (Supplementary Table S1 and Supplementary Figure S1). A subset of the putative Avrblb2-interacting proteins were calmodulins (SlCaM3 [Solyc10g077010], SlCaM4 [Solyc11g072240] and SlCaM5 [Solyc12g099990]) (Figure 1B). Due to the importance of calmodulin and calcium signaling in numerous plant microbe interactions, we conducted detailed investigations of the interaction of Avrblb2 with CAMs. Although calmodulins are highly conserved proteins in eukaryotes (Friedberg and Rhoads, 2001), there are variations at amino acid levels. Initially diverse CaMs; SlCaM3, NbCaM1, 3, 4, AtCaM2 and 6 were tested for their interaction with Avrblb2. For further Avrblb2- CaMs interaction studies, NbCaM3 (here after CaM3) and NbCaM1 (here after CaM1) were selected because these were the most divergent (19.2%) CaMs. After confirmation that Avrblb2 interacts with diverse CaMs, CaM3 was used for all other assays.

The Y2H results were verified *in vivo* using BiFC analyses in the *Arabidopsis* mesophyll protoplasts using YFPn-CaM3 and YFPc-Avrblb2 fusion proteins (Figure 1C). These analyses also revealed that Avrblb2 associates with CaM primarily at the plasma membrane with some cytoplasmic and nuclear localization (Figure 1C). The Avrblb2-CaM BiFC interactions, were also verified in *N benthamiana* leaves using *Agrobacterium*-mediated transient expression assays (Figure 1D).

### Avrblb2 Interacts With Calmodulin in a Ca^2+^-Dependent Manner

**FIGURE 2:**
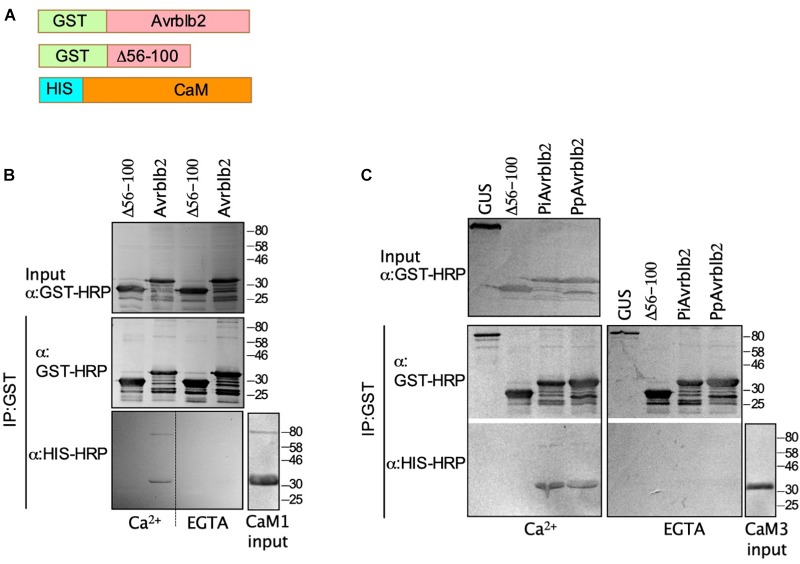
The *P. infestans* and *P. parasitica* Avrblb2 interact with calmodulin in a Ca^2+^–dependent manner. **(A)** Schematics of 6x His-tagged CaM and GST-tagged Avrblb2 homologs: *P. parasitica* (PpAvrblb2), *P. infestans* (PiAvrblb2), and the *P. infestans* truncated Avrblb2 (Δ56-100). (B and C) Immunoblots of *in vitro* co-IP assays showing: the interaction of GST-PiAvrblb2 with HIS-CaM1**(B)** and the interaction of both GST-PiAvrblb2 and GST-PpAvrblb2 with HIS-CaM3 **(C)** in the presence of Ca^2+^ but not EGTA, a Ca^2+^-chelator. Both negative controls Δ56-100 and GUS didn’t show any CaM pull down indicating specificity of Avrblb2-CaM interactions. All fusion proteins were expressed in E. coli, inputs and IP were run on duplicate 12% SDS polyacrylamide gels and transferred to PVDF membranes. Expression of GST-Avrblb2 and His-CaM fusion proteins was detected using an Anti-GST-HRP and Anti-His-HRP antibodies, respectively.

CaMs bind their target proteins either in a Ca^2+^-dependent or independent manner (Kaleka et al., 2012). To determine if Avrblb2 interacts with diverse CaMs in Ca^2+^-dependent manner and to further validate the Y2H and BiFC analyses, we conducted *in vitro* pulldown assays using bacterially expressed GST tagged Avrblb2 and 6×His-tagged CaM1 and CaM3 using anti-GST beads (Figure 2A). These analyses showed that Avrblb2 interacted with both CaMs in the presence of Ca^2+^ but not EGTA, a Ca^2+^ chelator (Figure 2B,C). Negative controls consisting of GST-tagged GUS and a truncated version of GST-Avrblb2 (Δ56-100) did not interact with CaMs, showing that the interaction of Avrblb2 with calmodulins is specific (Figure 2B,C).

### Calmodulin Interacts With Divergent Avrblb2 Homologs

**FIGURE 3:**
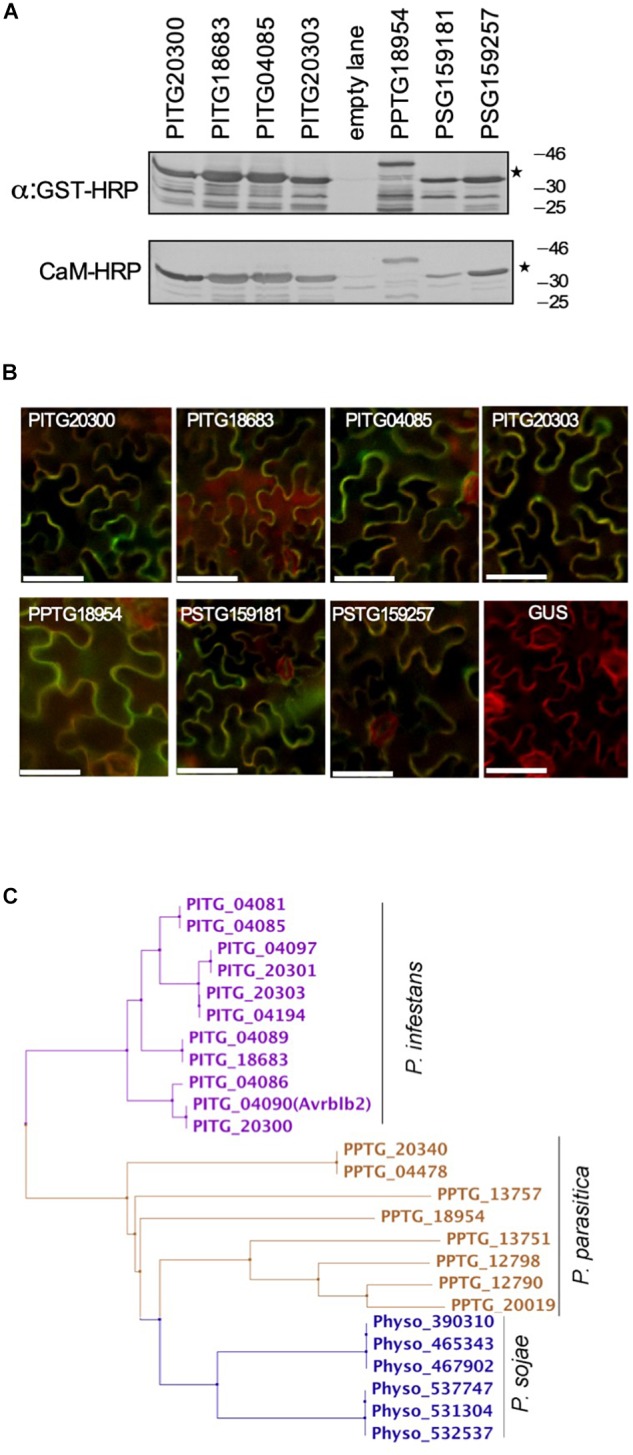
Most of the divergent Avrblb2 homologs interact with calmodulin in a Ca^2+^-dependent manner. **(A)** *In vitro* CaM-HRP overlay assay show that GST-tagged divergent Avrblb2 homologs from three *Phytophthora* species, *P. infestans* (PITG20300, PITG18683, PITG04085, and PITG20303), *P. parasitica* (PPTG18954) and *P. sojae* (PSG159181 and PSG159257) interact with calmodulin (CaM:HRP). GST-Avrblb2 fusion proteins, expressed in E. coli, were run on duplicate 12% SDS polyacrylamide gels and transferred to PVDF membranes. Expression of GST-Avrblb2 fusion proteins was detected using an Anti-GST-HRP antibody (Upper panel) and CaM-binding of GST-Avrblb2 proteins was assessed using CaM-binding overlay assays using AtCaM2::HRP antibody. Exact expected bands of both CaM and Avrblb2 are marked by star. **(B)** Transient BiFC analyses show that divergent Avrblb2 homologs interact with calmodulin *in vivo*. The indicated Avrblb2 homologs fused to YFPn were co-expressed with CaM3-YFPc in *N. benthamiana* leaves stably expressing a membrane-localized red fluorescent protein marker (RFP::PMRK). Green/Yellow fluorescence indicates BiFC of Avrblb2 with calmodulin. Scale bar represents 50 μm. **(C)** Phylogeny of Avrblb2 homologs. Amino acids 34 (48–81) were aligned using ClustalW and phylogenetic tree was constructed using the Maximum Likelihood (ML) method with 1000 bootstrap replication (Tamura and Nei, 1993; Tamura et al., 2011).

The *P. infestans* genome encodes at least 11 Avrblb2 homologs. Amino acid sequence comparison shows that Avrblb2 homologs are highly similar at the N-terminus but are divergent at the C-terminus (Supplementary Figure S2). BLAST search analyses of sequenced *Phytophthora* spp. genomes showed that *P. parasitica* and *P. sojae* also carry at least eight and two Avrblb2 homologs, respectively (Figure 3C and Supplementary Figure S2). To test if divergent Avrblb2 homologs also interact with CaM3, we first tested the interaction of the *P. parasitica* Avrblb2 homolog (PpAvrblb2) with CaM3. BiFC analyses in *N. benthamiana* revealed that the PpAvrblb2 also interacted with CaM3 at plasma membrane (Figure 1D). Pull down assay of bacterially expressed GST tagged PpAvrblb2 and 6xHis-tagged CaM3 using anti-GST beads in the presence and absence of Ca2^+^ revealed that the interaction of PpAvrblb2 with CaM3 is also Ca^2+^-dependent (Figure 2C).

Phylogenetic analyses of the C-terminal effector domain of the *P. infestans* Avrblb2 homologs resulted in four major groups (Figure 3C). One representative member from each of these groups, PITG20300, PITG18683, PITG04085, and PITG20303, were also tested for binding to calmodulin in a CaM-HRP overlay assay. As is shown in Figure 3A, all Avrblb2 homologs also bound calmodulin. Similarly, the *P. parasitica* homolog (PPTG18954) also bound calmodulin (Figure 3A). The *P. sojae* genome appears to harbor six hypothetical Avrblb2 genes, which code for two Avrblb2 homologous proteins^2^. These two *P. sojae* Avrblb2s are highly divergent from the *P. infestans* and *P. parasitica* Avrblb2s (Supplementary Figure S2). *In vitro* assays, each of these two *P. sojae* Avrblb2 homologs also bound calmodulin (Figure 3A). Using BiFC analyses, the interaction of all these Avrblb2 homologs to calmodulin was also verified *in vivo* in *N. benthamiana* leaves (Figure 3B). Overall, these analyses suggest that calmodulin interact with divergent Avrblb2 homologs.

### The Calmodulin-Interaction Domain Is Located in the Effector Domain of Avrblb2

**FIGURE 4:**
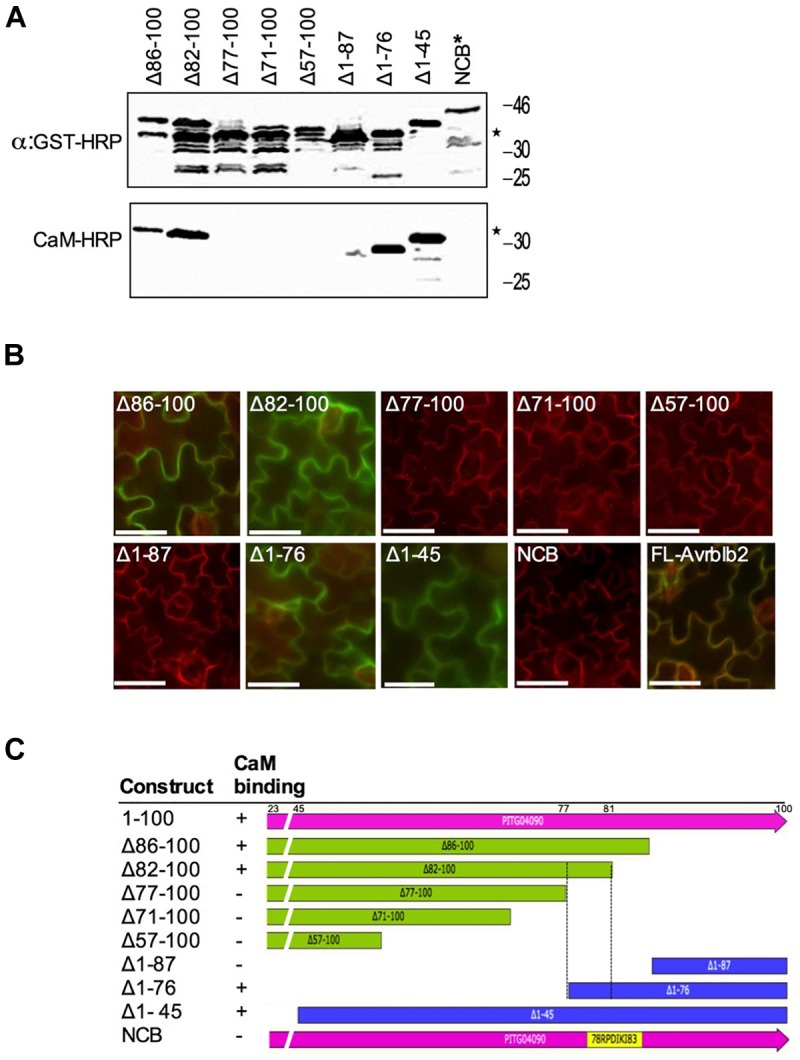
Calmodulin-binding site mapping of the *P. infestans* Avrblb2. **(A)** CaM-overlay assays of PiAvrblb2 mutants performed to find out CaM binding region show that CaM binding activity was lost when a five amino acids C-terminal region from position 78 to 82 was deleted. Mutant NCB indicated by ^∗^ in which all five amino acids (78–82) were replaced with Alanine also didn’t show any CaM binding. Expression of GST-Avrblb2 fusion proteins was detected using an Anti-GST-HRP antibody (Upper panel) and CaM-binding of PiAvrblb2 mutant proteins was assessed using CaM-binding overlay assays using AtCaM2::HRP antibody. Exact expected bands of both CaM and Avrblb2 are marked by star. **(B)** *In planta* BiFC assays showing interaction of PiAvrblb2 mutants fused to YFPn with CaM fused to YFPc. Just like CaM-overlay assay, mutants having intact CBD (77–81): Δ86–100, Δ82–100, Δ1–76, Δ1–45 and full length Avrbl2 show interaction with CaM and give green/yellow fluorescence. All others including NCB didn’t show interaction. BiFC analyses were carried out using transgenic *N. benthamiana* leaves expressing a membrane-localized red fluorescent protein marker (RFP::PMRK). Scale bar represents 50 μm. **(C)** Schematics of N-terminal and C-terminal deletion constructs of PiAvrblb2 and their binding with CaM. Boundaries of CBD are shown by black dotted lines.

To identify CaM binding domain (CBD) in Avrblb2, we made a series of C- and N-terminal deletion mutants of the PiAvrblb2 protein (Figure 4C). These deletion mutants were tested *in vitro* for interaction with calmodulin using calmodulin binding overlay assays. These analyses revealed that deletion mutants lacking C-terminal aa 77–100 (Δ77–100) or N-terminal aa 1–87 (Δ1–87) lost calmodulin binding activity, whereas constructs longer than these two these two mutants retained calmodulin-binding activity (Figure 4A,C). These results were verified *in vivo* BiFC analyses (Figure 4B). To make sure that only this region is sufficient for calmodulin binding, we mutated all five amino acids (aa 78–82) in this region to alanine, which completely abolished its interaction with calmodulin (Figure 4A,B). All together, these analyses suggest that amino acids 78–82 in the C-terminal region of Avrblb2 are important for binding to CaM (Figure 4C).

**FIGURE 5:**
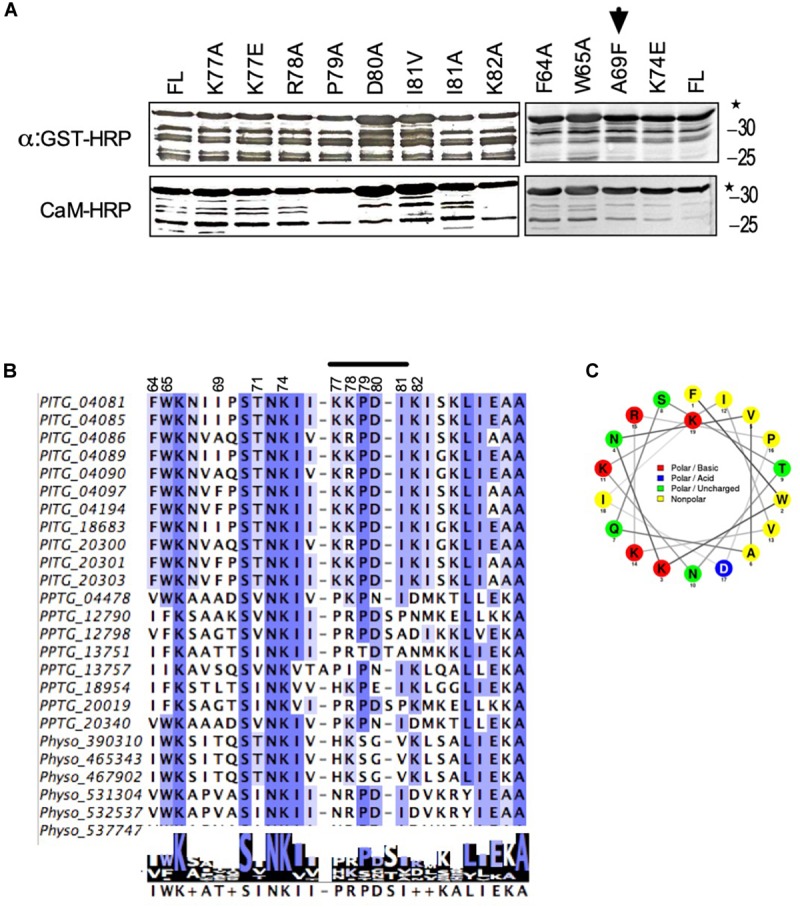
Identification of amino acids in avrblb2, which are critical for interaction to calmodulin. **(A)** Immunoblot showing CaM-overlay assays of PiAvrblb2 variants mutated at potential anchor sites (left panels) and variants having polymorphism at position 69 (right panel). All these Avrblb2 variants showed CaM binding activity depicting that none of these amino acids play any role in CaM binding. A69F indicated by arrow is Avrblb2 variant that is not recognized by cognate *R* gene Rpi-blb2. All Avrblb2 variants were GST tagged and their expression was detected using an Anti-GST-HRP antibody (Upper panel) and CaM-binding of GST-Avrblb2 proteins was assessed using CaM-binding overlay assays using AtCaM2::HRP antibody. Exact expected bands of both CaM and Avrblb2 are marked by star. **(B)** Amino acid (64–90) alignment of Avrblb2 homologs of *P. infestans, P. parasitica* and *P. sojae* to visualize sequence variations and to identify potential anchor sites. and at position 69, in CBD. Positions 64, 65, 74 and 79 were identified as potential anchor sites. Position 69 is highly polymorphic. CBD show substantial variations in different Avrblb2 homologs (Supplementary Figure S3). **(C)** Wheel projection diagram of Avrblb2 to show CBD.

CBD domains usually contain hydrophobic anchor amino acids, which occur in different configurations, usually 1–8–14, 1–5–10 in different CaM binding proteins (Tidow and Nissen, 2013). Based on this information and our results from deletion analyses, we identified amino acids at position 64, 65, 74, and 79 as potential anchor sites. To determine if any of these amino acids are responsible for interaction with CaM, we changed each one of them to alanine (A) one by one and tested them for their interaction to CaM. Calmodulin-binding overlay assays showed, that none of these amino acids affected calmodulin binding suggesting that these amino acids are not solely responsible for interaction with calmodulin (Figure 5A, right panel). Absence of interaction was not due to lack of protein expression as all these mutants were expressed very well (Figure 5A, upper panels). Since deletion analyses delineated CBD to amino acids 78 – 82, using alanine scanning, we mutated single amino acids in this region to alanine and tested their interaction with calmodulin. All mutants bound CaM (Figure 5A) but when all five amino acids from position 78 to 82 were changed to alanine the interaction was broken (Figure 4A, (NCB) marked by ^∗^), suggesting that different amino combinations between amino acids 78 – 82 contribute to calmodulin binding.

### The Positively Selected Amino Acid at Position 69 in Avrblb2 Does Not Coincide With Calmodulin Binding Domain

The amino acid position 69 in Avrblb2 is highly polymorphic and affects its recognition by Rpi-blb2 (Oh et al., 2009). To investigate whether this position affects CaM binding with Avrblb2 we tested different variants and found that regardless of amino acid type at position 69, all Avrblb2 homologs (PITG20300 with A69, PITG18683 with I69, PITG04085 with I69, and PITG20303 with F69) bound equally well to calmodulin (Figure 5A). To further verify we changed A69 to F69 in PITG04090, which is recognized by Rpi-blb2 and which we have used as template for constructing deletion and single amino acid mutants. Calmodulin-binding assays showed that the PITG04090-A69F mutant also retained calmodulin binding (Figure 5A arrow) suggesting that aa 69-dependent Rpi-blb2 activation by Avrblb2 is independent of calmodulin binding.

### Calmodulin-Binding to Avrblb2 Is Required for Rpi-blb2-Dependent Cell Death

**FIGURE 6:**
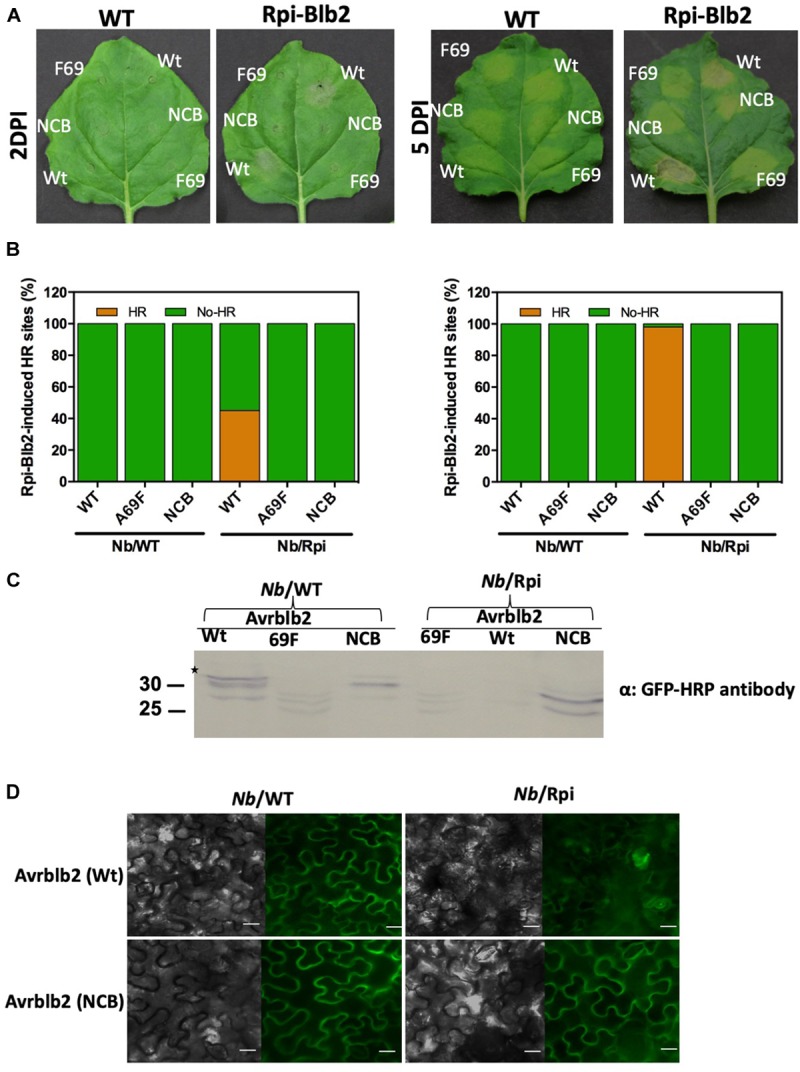
The Avrblb2/Rpi-blb2-induced cell death is dependent on calmodulin binding to Avrblb2. **(A)**. Representative *N. benthamiana* leaves, 2 and 5 days post Agro-infiltration of YFP tagged WT-Avrblb2 (Wt), Avrblb2-NCB (NCB) and Avrblb2-A69F (F69). HR symptoms are clearly visible at spots infiltrated with WT-Avrblb2 only in Rpi-blb2 containing transgenic *N. benthamiana* (NB/Rpiblb2) leaves both at 2 and 5 days whereas Avrblb2-NCB just like the negative control (Avrblb2-A69F) didn’t show any signs of HR even at 15 dpi. As expected, no HR spots were seen on Nb/WT leaves both at 2 and 15 dpi (Supplementary Figure S4). **(B)** Bar graph of infiltrated sites showing HR after 2 days, and 15 days of inoculation. Our non-calmodulin binding Avrblb2 mutant (NCB) didn’t show any HR spot compared to positive control, WT-Avrblb2 that showed around 45% visible HR spots at 2 dpi and around 100% strong HR at 15 dpi. **(C)** Western blots of proteins extracted from Nb/WT and Nb/Rpi leaves at 1 dpi. In Nb/WT (left three lanes) all three proteins Wt, F69 and NCB are expressed whereas in Nb/Rpi expression of F69 and NCB was comparable to their expression in Nb/WT but very faint band was observed for Wt Avrblb2 that indicates protein degradation even before visible HR. Anti-GFP-HRP antibody was used to detect expression of all fusion proteins. Note: BiFC construct of Avrblb2-A69F fused to YFPn was used as negative control, so smaller bands in F69 lanes are due to partial YFP. Exact expected bands of Avrblb2 mutants are marked by star. **(D)** Visualization of YFP tagged Wt and NCB in Nb/WT (left) and Nb/Rpi (right) leaves. No difference in plasma membrane localization of Wt and NCB was observed in both Nb/WT and Nb/Rpi leaves. Significant degradation of Nb/Rpi leaf spots infiltrated with Wt Avrblb2 was observed that indicates initiation of HR symptoms at 1 dpi. Scale bar represents 10 μm.

To determine whether CaM binding to Avrblb2 is essential for Rpi-blb2 mediated HR or not, we used Rpi-blb2 transgenic *N. benthamiana* to test our non-calmodulin binding mutant of Avrblb2 (NCB-Avrblb2) with mutated CBD (78–82aa). *Agrobacterium*-mediated infiltration of YFP tagged NCB-Avrblb2, WT-Avrblb2 and A69F-Avrblb2 (BiFC construct with nYFP) was done in both wild type and Rpi-blb2 transgenic *N. benthamiana* plants and HR symptoms were followed from 1 to 15 days post inoculation (dpi). WT-Avrblb2 was used as positive control and Avrblb2-A69F, which is not recognized by Rpiblb2 (Oh et al., 2009) was used as a negative control. Protein expression of these constructs was confirmed by looking for YFP under confocal microscope at 1 dpi (Figure 6D) and through western blot by using anti-GFP-HRP antibody (Figure 6C). As expected, none of the Avrblb2 variants caused any HR symptoms on WT *N. benthamiana* (Figure 6A). Consistent with previous results, WT Avrblb2 (PITG04090) did cause HR on transgenic *N. benthamiana* expressing Rpi-blb2 gene. HR symptoms were obvious at around 45% of spots infiltrated with WT-Avrblb2 only in Rpi-blb2 containing transgenic *N. benthamiana* (NB/Rpiblb2) leaves at 2 dpi and almost 100% HR spots were observed at 15 dpi (Figure 6B). Mild HR symptoms started to appear as early as 1 dpi and obvious cell degradation and disrupted YFP signals were observed under confocal microscope (Figure 6D). In contrast, NCB-Avrblb2 did not cause any HR just like A69F-Avrblb2 either 1 to 5 (Figure 6A,C) or even 15 dpi (Supplementary Figure S4). Moreover, NCB-Avrblb2 expression and plasma membrane localization in both WT and transgenic plants were exactly like WT-Avrblb2 in WT plants (Figure 6C). These findings suggest that calmodulin binding to Avrblb2 does play a role in its recognition by Rpi-blb2 receptor to induce cell death.

## Discussion

Oscillations in free Ca^2+^ concentration in response to biotic stress stimuli has been reported as a key early event and calmodulins are the primary sensors of calcium signaling pathways (Ranty et al., 2016). Several studies based on differential expression, overexpression or silencing of CaMs consistently showed their involvement in plant’s response to pathogens (Do Heo et al., 1999; Takabatake et al., 2007; Choi et al., 2009; Ali et al., 2013; Cheval et al., 2013). However, direct physical interaction between pathogen effectors and host calmodulins is reported very recently in case of a *Pseudomonas syringae* effector HopE1 and a *P. infestans* effector SFI5 (Guo et al., 2016; Zheng et al., 2018). Here we report the interaction of Avrblb2, a core RXLR effector of *Phytophthora* species, with calmodulin. The expression of Avrblb2 is specifically induced during the early biotrophic stages of infection (Brasier, 2009; Haas et al., 2009) and the Avrblb2-CaM interaction could be speculated as an early event of host-pathogen cross talk to modulate Ca^2+^ dependent-defense signaling during the early stages of infection.

Binding of calmodulin to its targets could be either Ca^2+^-dependent or -independent (Rhoads and Friedberg, 1997). Upon binding Ca^2+^, CaMs undergo conformational changes to bind its downstream targets (Kilhoffer et al., 1988). Consistent with this fact, activation of CaMs to interact with Avrblb2 also requires Ca^2+^. This is also consistent with the observation that the interaction of *P. syringae* effector HopE1 and *P. infestans* effector SFI5 with calmodulin is also Ca^2+^-dependent (Guo et al., 2016; Zheng et al., 2018).

Avrblb2s belong to a polymorphic gene family reported to have undergone diversifying selection (Oh et al., 2009). Multiple Avrblb2 paralogs have been found in the genomes of different *Phytophthora* spp. (Oh et al., 2009; Oliva et al., 2015). Virulence function of one *P. infestans* Avrblb2 homolog (PexRD40) was associated with preventing the secretion of a plant defense protease (C14) to the apoplast apparently to protect intercellular growth of *Phytophthora* (Bozkurt et al., 2011). We found that divergent Avrblb2 homologs of *P. infestans, P. parasitica* and *P. sojae* interact with calmodulins suggesting that the Avrblb2-CaM interactions are general, and they could be playing a conserved role in plant-*Phytophthora* interactions. CaMs are the calcium sensors that have multiple downstream protein targets to operate diverse pathways, combined outcomes of which results in specific response to particular stimuli. Thus, Avrblb2-CaM interaction could be a key for the pathogen to intervene multiple pathways. Moreover, reports on an effector targeting multiple diverse host proteins are scarce.

Avrblb2s are about 100 amino acid (aa) long modular proteins consisting of an N-terminal signal peptide, a RXLR motif and a 50 aa C-terminal effector domain (Oh et al., 2009; Oliva et al., 2015). Calmodulin-binding domains (CBDs) are small 5–20 amino acids long and highly sequence divergent and are thus not easy to predict using sequence-based predictions (Tidow and Nissen, 2013). Deletion mutant analyses of Avrblb2 indicate that the CBD resides between amino acids 77 and 82 in the effector domain of Avrblb2. Sequence specificity of CBD was tested by replacing each one of five CBD residues with alanine but none of those five mutants abolished Avrblb2-CAM binding. However, replacement of whole CBD with alanine completely abolished CAM binding activity suggesting that multiple amino acids contribute to calmodulin binding in this CBD region. This observation is unique as many of the characterized CBDs have at least one essential amino acid that when mutated completely abolishes calmodulin binding.

*Rpi-blb2*, the cognate *R* gene of Avrblb2 is well known to confer durable broad spectrum resistance against *P. infestans* and is being widely used for developing late blight resistant commercial potato cultivars (Oliva et al., 2015; Orbegozo et al., 2016). Avirulence eliciting region of *P. infestans* Avrblb2 homologs required for the activation of Rpi-blb2 resides in highly conserved 34 amino acid region with a polymorphic residue at position 69. Among different Avrblb2 aa 69 variants occurring in natural *P. infestans* populations, variants with only Phe-69 (A69F) failed to induce Rpi-blb2 mediated HR (Oh et al., 2009). We found all aa 69 polymorphic variants including A69F invariably bind CaM suggesting that calmodulin binding to Avrblb2 is independent of aa 69 polymorphism. Protein models for WT and A69F Avrblb2s showed that although very close to Avrblb2-CaM interaction site, A69 is out of CaM binding domain. In protein model amino acid 69F showed a bulge in Avrblb2 A69F variant that we speculate doesn’t interferes Avrblb2-CaM binding but do affect its recognition by Rpiblb2.

The Avrblb2 CBD identified in our studies is located within the HR-eliciting domain of Avrblb2 prompting us to hypothesize that calmodulin binding could have a role in the Rpi-blb2 mediated HR. Our results indicate that the non-calmodulin-binding Avrblb2-NCB failed to induce any HR in Rpi-blb2 expressing *N. benthamiana* plants, suggesting that Rpi-blb2 induced HR depends upon CaM binding with Avrblb2. Based on these observations, we speculate that CaM serve as a guardy or decoy, which is monitored by Rpi-blb2 for binding to Avrblb2 to induce HR. This is in contrast to the Avrblb2/C14 interaction, which was reported to not be involved in the recognition of Avrblb2 by Rpi-blb2 (Bozkurt et al., 2011). Currently, there is no evidence for direct binding of calmodulin to any canonical NB-LRR type R proteins. However, direct interaction of CaM with the barley MLO, a non-NB-LRR type recessive resistance gene, or more accurately a recessive susceptibility *S* gene, was shown to play an essential role in regulating powdery mildew resistance (Kim et al., 2002).

Rpi-blb2 mediated cell death in response to PiAvrblb2 has been shown to require SGT1, a eukaryotic co-chaperone widely reported to be involved in both PTI and ETI associated cell death responses (Abascal et al., 2014; Liu et al., 2016). The *P. infestans INF1* elicitor-induced HR also require SGT1 (Abascal et al., 2014). Since *SGT1* is involved in Ca^2+^ signaling-dependent HR induction in pepper plants in response to *P. capsici INF1* elicitor (Liu et al., 2016), HR induction by Rpi-blb2 in response to Avrblb2-CaM interaction might have some direct or indirect link with SGT1 pathway.

Localization of Avrblb2 is important for its virulence function. Consistent with the previous findings we observed plasma membrane localization of Avrblb2 *in planta* (Bozkurt et al., 2011). Moreover, we found that Avrblb2 interacts with CaM primarily at the plasma membrane, which is also consistent with the localization of Avrblb2-C14 interaction (Bozkurt et al., 2011). Calmodulins are essential components of Ca^2+^ signaling pathways, which are involved in coupling numerous environmental stimuli including pathogens to whole plant physiology (Reddy et al., 2011; Ranty et al., 2016). Interaction of divergent Avrblb2 homologs with calmodulin suggests a generalized and conserved function for the Avrblb2-CaM interaction in interrupting multiple host signaling pathways to modulate defense responses. Ca^2+^ signatures are very specific in response to different biotic stimuli and vary greatly in response to compatible/incompatible interactions (Reddy et al., 2011). In the light of all these findings we suggest that calmodulin-dependent Ca^2+^ signaling plays a critical role in determining virulence or avirulence activity of Avrblb2 in host cells.

## Author Contributions

GA conceived and designed the experiments, and revised and approved the final version of the manuscript. ZN, SB, and GA performed the experiments. ZA and GA wrote the manuscript.

## Conflict of Interest Statement

The authors declare that the research was conducted in the absence of any commercial or financial relationships that could be construed as a potential conflict of interest.

## Footnotes

1 http://invitrogen.com

2 http://eumicrobedb.org/eumicrobedb/index.php

## Supplementary Material

FIGURE S1 Y2H screening results.

FIGURE S2 Amino acid alignment of Avrblb2 homologs.

FIGURE S3 Wheel projections of Avrblb2 homologs.

FIGURE S4 HR response of WT and NCB Avrblb2 at 15 dpi.

TABLE S1 Putative Avrblb2 interacting proteins identified in Yeast Two hybrid screens.

TABLE S2 Primers used for cloning of Avrblb2 effectors genes from *P. infestans, P. parasitica* and *P. sojae.*
