# Supplementary figures and images for: The Phytophthora RXLR Effector Avrblb2 Modulates Plant Immunity by Interfering With Ca2+ Signaling Pathway
Source: Front Plant Sci. 2019 Mar 28;10:374. doi: 10.3389/fpls.2019.00374 (PMC6447682; doi:10.3389/fpls.2019.00374)

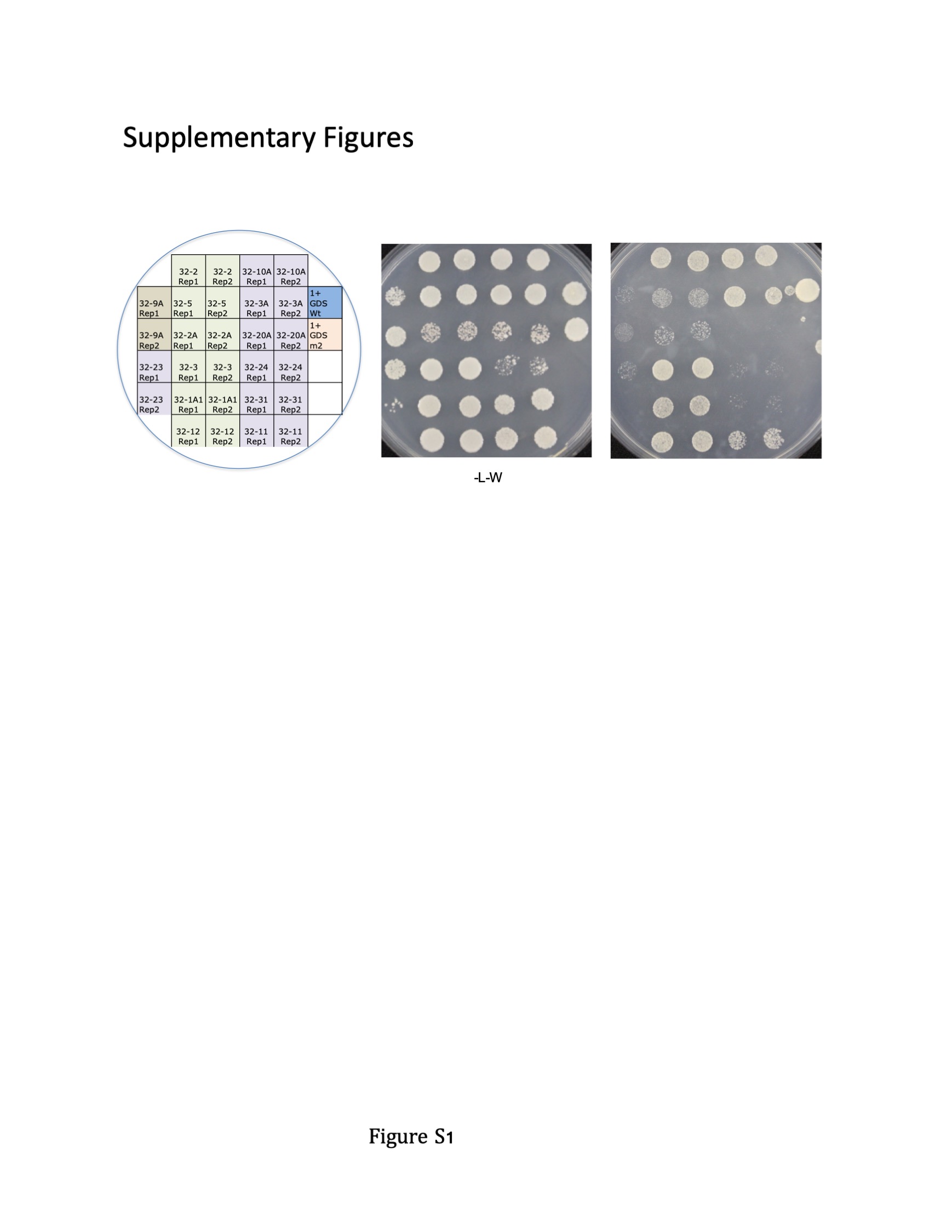

Supplement: FIGURE S1 — Y2H screening results. [file Image_1.JPEG]

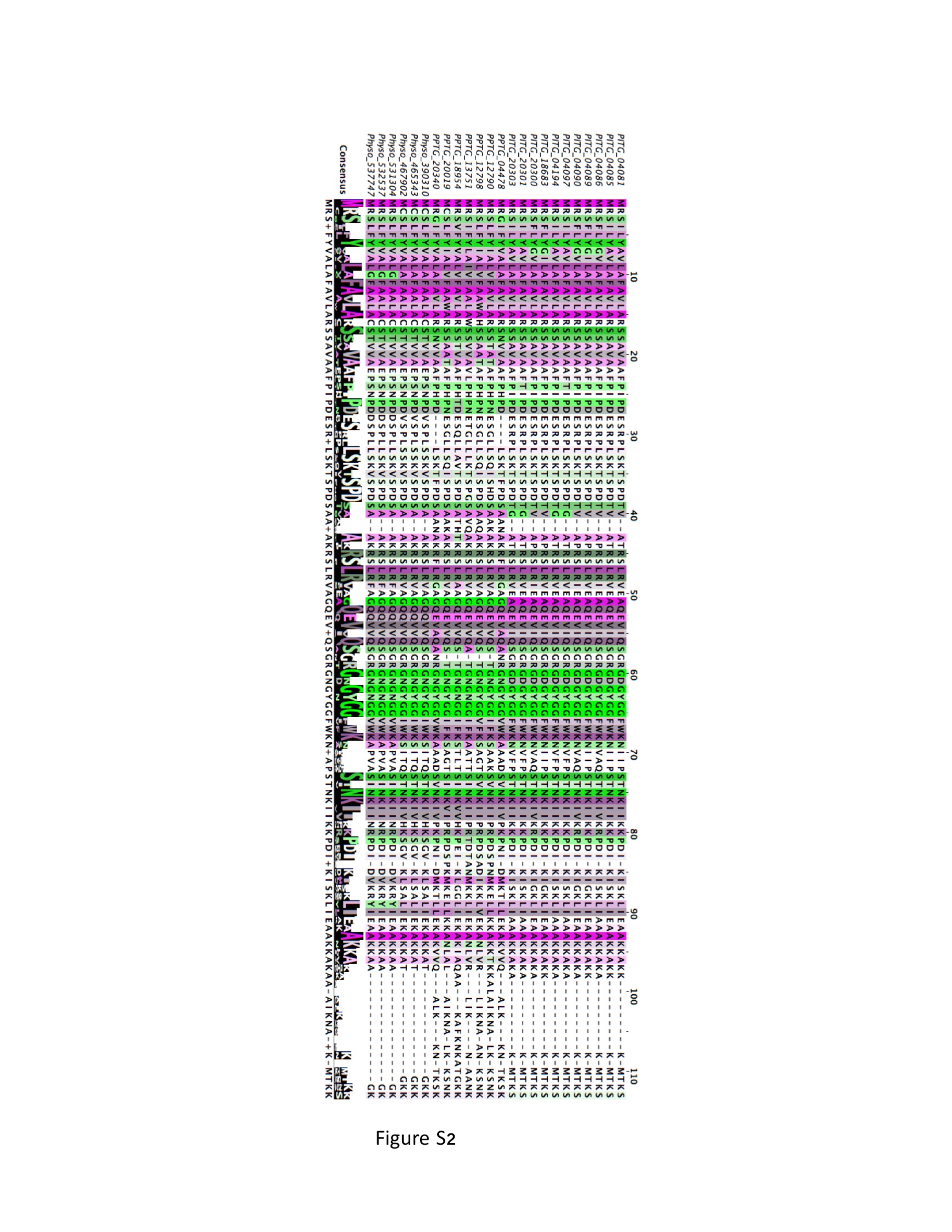

Supplement: FIGURE S2 — Amino acid alignment of Avrblb2 homologs. [file Image_2.JPEG]

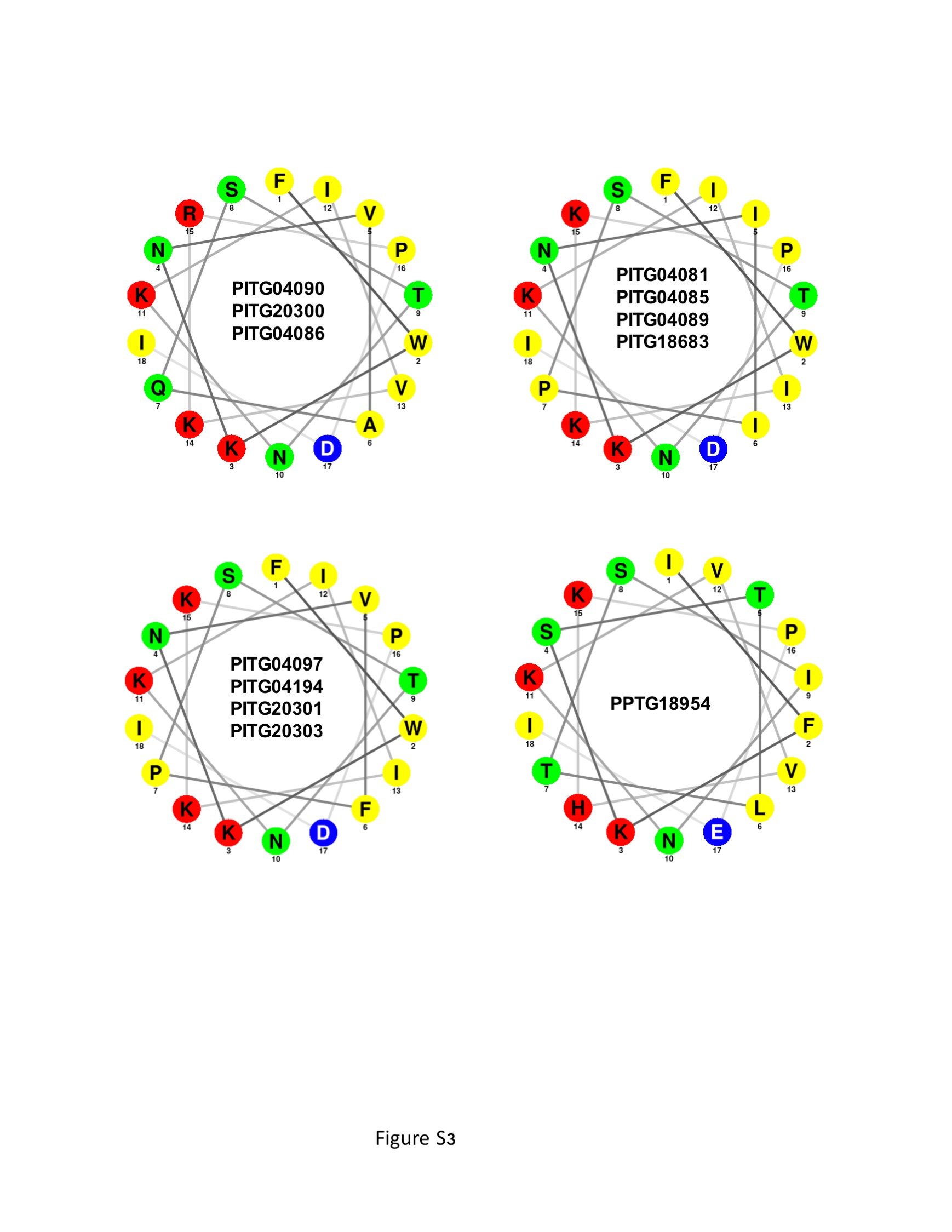

Supplement: FIGURE S3 — Wheel projections of Avrblb2 homologs. [file Image_3.JPEG]

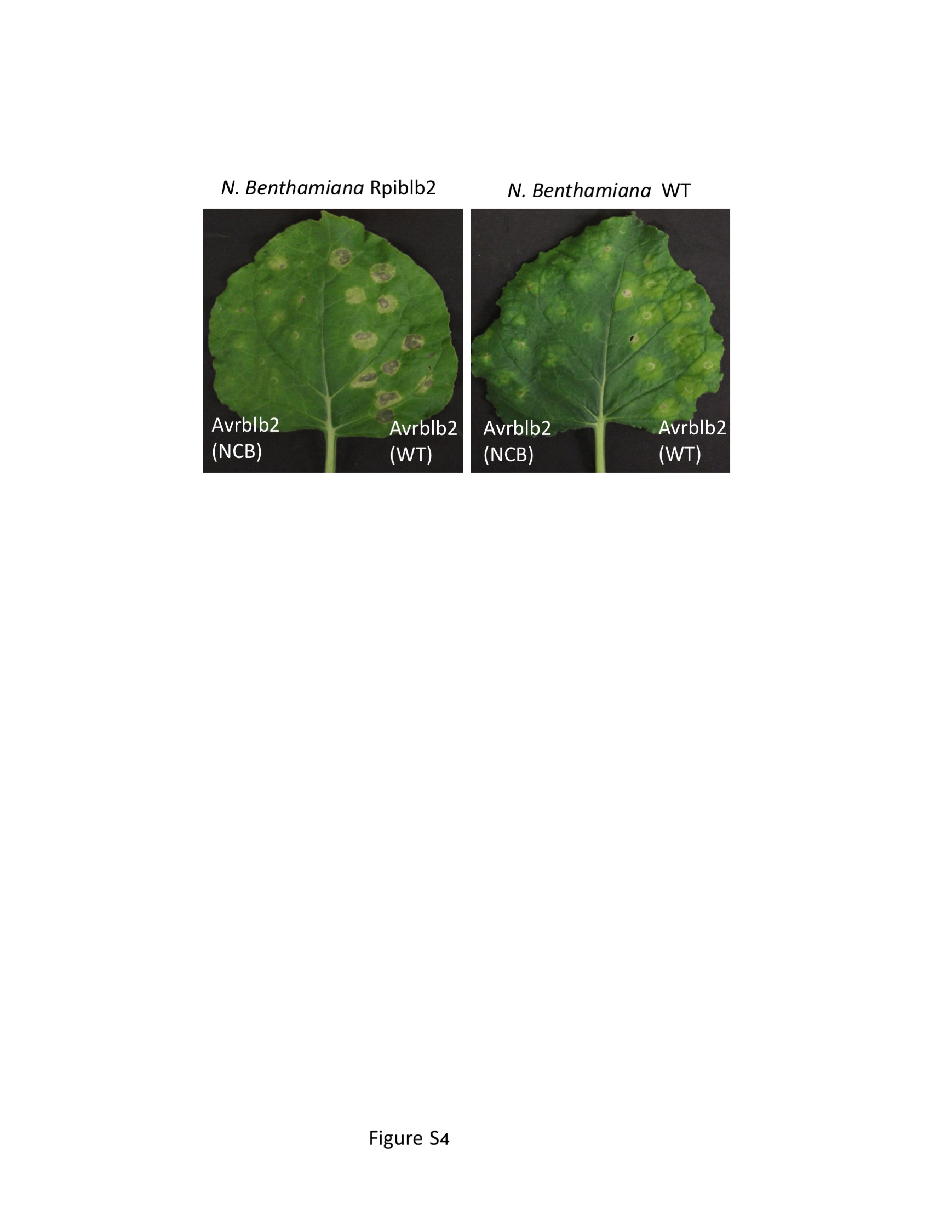

Supplement: FIGURE S4 — HR response of WT and NCB Avrblb2 at 15 dpi. [file Image_4.JPEG]
