# Supplementary material for: The Phytophthora RXLR Effector Avrblb2 Modulates Plant Immunity by Interfering With Ca2+ Signaling Pathway
Source: Front Plant Sci. 2019 Mar 28;10:374. doi: 10.3389/fpls.2019.00374 (PMC6447682; doi:10.3389/fpls.2019.00374)
Supplement: TABLE S1 — Putative Avrblb2 interacting proteins identified in Yeast Two hybrid screens. [file Data_Sheet_1.PDF]

**Table S1: Putative Avrblb2 interactng proteins identified in Yeast Two hybrid screens**

| Prey Clone# | Accession | Description | Total score | Query coverage | E-value | Max identity |
|---|---|---|---|---|---|---|
| 32-2 | XP_004250331.1 | Selenoprotien K | 97.8 | 43% | 2.00E-22 | 99% |
| 32-3 | YP_008563085.1 | Photosystem II protein Z | 43.9 | 20% | 4.00E-04 | 100% |
| 32-1A1 | XP_004240442.1 | FRIGIDA-like protein 3 | 662 | 92% | 0 | 93% |
| 32-12 | XP_004230773.1 | Putative thioredoxin m2 | 332 | 52% | 3.00E-111 | 96% |
| 32-10A | NP_001234007.1 | 14-3-3 protein 4 | 49.7 | 20% | 3.00E-05 | 100% |
| 32-3A | NP_001308423.1 | Calmodulin [SlCaM3 (Solyc10g077010), SlCaM4(Solyc11g072240), SlCaM5 (Solyc12g099990); | 302 | 63% | 2.00E-105 | 100% |
| 32-11A | NP_001233881.1 | ArcA2 protein | 697 | 95% | 0 | 99% |
