# Supplementary material for: The Phytophthora RXLR Effector Avrblb2 Modulates Plant Immunity by Interfering With Ca2+ Signaling Pathway
Source: Front Plant Sci. 2019 Mar 28;10:374. doi: 10.3389/fpls.2019.00374 (PMC6447682; doi:10.3389/fpls.2019.00374)
Supplement: TABLE S2 — Primers used for cloning of Avrblb2 effectors genes from P. infestans, P. parasitica and P. sojae. [file Data_Sheet_2.PDF]

Table S2: List of primers used in this study

Construct	Froward primer	Sequence	Reverse primer	Sequence
PITG04090	PITG_04090F	caccatgTTCCCAATCCCCGACGAGTCTCGC	PITG_04090R	GGACTTCGTCATTTTTGCTTTTGCCTTCTTGGCC
PITG04090[Δ57-100]	XhoAttL2-InvrsF	tgactcgagggtgacCCAGCTTTCTTGTACAAAGT	PITG04090-CdelG56r	gtcaccctcgagtcaGCCGCTCTGAATAACTTCTTGG
PITG04090[Δ86-100]	XhoAttL2-InvrsF	tgactcgagggtgacCCAGCTTTCTTGTACAAAGT	PITG04090-CdelK85r	gtcaccctcgagtcaTTTGCTTATCTTGATATCCGGCC
PITG04090[Δ77-100]	XhoAttL2-InvrsF	tgactcgagggtgacCCAGCTTTCTTGTACAAAGT	PITG04090-CdelK76r	gtcaccctcgagtcaGACGATCTTGTTAGTACTCTG
PITG04090[Δ83-100]	XhoAttL2-InvrsF	tgactcgagggtgacCCAGCTTTCTTGTACAAAGT	PITG04090-CdelK82r	gtcaccctcgagtcaGATATCCGGCCTCTTGACG
PITG04090[Δ71-100]	XhoAttL2-InvrsF	tgactcgagggtgacCCAGCTTTCTTGTACAAAGT	PITG04090-CdelS71r	gtcaccctcgagtcaCTGGGCTACGTTTTTCCAG
PITG04090[Δ1-88]	PITG04090-NdelK88f	gccttcaccatggctGCGGCCAAGAAGGCAAA	NcoIAttL1-InvrsR	agccatggtgaaggcGGCCGCGGAGCCTG
PITG04090[Δ1-45]	PITG04090-NdelE48f	gccttcaccatggctGAGGCCCAAGAAGTTATTCAGAG	NcoIAttL1-InvrsR	agccatggtgaaggcGGCCGCGGAGCCTG
PITG04090[Δ1-76]	PITG04090-NdelK77f	gccttcaccatggctAAGAGGCCGGATATCAAGATAAG	NcoIAttL1-InvrsR	agccatggtgaaggcGGCCGCGGAGCCTG
PITG04090[77-87]	XhoAttL2-InvrsF	tgactcgagggtgacCCAGCTTTCTTGTACAAAGT	PITG04090-87AArInfs	gtcaccctcgagtcaAAGTTTGCTTATCTTGATATCCGG
PITG04090[X78Ax5]	78Ax5infsF	GCAGCCGCCGCTGCAGCTAGCAAACTTATCGCG	78Ax5infsR	TGCAGCGGCGGCTGCCTTGACGATCTTGTTAGTACTCTG
PITG04090[X77Ax9]	77Ax9infsF	GCAGCCGCCGCTGCAGCTGCCGCACTTATCGCGGCGG	77Ax9infsR	TGCAGCGGCGGCTGCGGCGACGATCTTGTTAGTACTCTG
PITG04090[K77A]	K77AinfuseF	agcactaacaagatcgtcGCgaggccggatatcaagata	V76infuseR	GATCTTGTTAGTgCTCTGGGCTAC
PITG04090[K77E]	K77EinfuseF	agcactaacaagatcgtcGagaggccggatatcaagata	V76infuseR	GATCTTGTTAGTgCTCTGGGCTAC
PITG04090[R78A]	R78AinfuseF	agcactaacaagatcgtcaagGCgccggatatcaagata	V76infuseR	GATCTTGTTAGTgCTCTGGGCTAC
PITG04090[P79A]	P79AinfuseF	agcactaacaagatcgtcaagaggGCCgatatcaagata	V76infuseR	GATCTTGTTAGTgCTCTGGGCTAC
PITG04090[D80A]	D80AinfuseF	agcactaacaagatcgtcaagaggccggCtatcaagata	V76infuseR	GATCTTGTTAGTgCTCTGGGCTAC
PITG04090[I81A]	I81AinfuseF	agcactaacaagatcgtcaagaggccggatGCcaagata	V76infuseR	GATCTTGTTAGTgCTCTGGGCTAC
PITG04090[I81V]	I81VinfuseF	agcactaacaagatcgtcaagaggccggatGtcaagata	V76infuseR	GATCTTGTTAGTgCTCTGGGCTAC
PITG04090[K82A]	K82AinfuseF	agcactaacaagatcgtcaagaggccggatatcGCgata	V76infuseR	GATCTTGTTAGTgCTCTGGGCTAC
PITG04090[I83A]	I83AinfuseF	agcactaacaagatcgtcaagaggccggatatcaagGCaAG	V76infuseR	GATCTTGTTAGTgCTCTGGGCTAC
PITG04090[F64A]	[3-3]F64A-infuseF	gcCTGGAAAAACGTAGCCCAGAGTACaAACAAG	[3-3]F64A-infuseR	TACGTTTTTCCAGgcCCCACCATATCCG
PITG04090[W65A]	[3-3]W65A-infuseF	gcGAAAAACGTAGCCCAGAGTACaAACA	[3-3]W65A-infuseR	TACGTTTTTCgcGAACCCACCATATCC
PITG04090[A69F]	[3-3]A69F-infuseF	ttCCAGAGTACaAACAAGATCGTCAAGAG	[3-3]A69F-infuseR	GTTtGTACTCTGGaaTACGTTTTTCCAGAAC
PITG04090[K74E]	[3-3]K74E-infuseF	aAACgAGATCGTCAAGAGGCCGG	[3-3]K74E-infuseR	TTGACGATCTcGTTtGTACTCTGGGC
PITG04090[71TNKI75VEET]	[3-3]71TNKIinfuseF	gtagaagaaactgtcAAGAGGCCGGATATCAAGATAAG	[3-3]71TNKIinfuseR	gacagtttcttctacACTCTGGGCTACGTTTTTCC
PPTG20340;PPTG04478	PPTG_20340F	caccatgCCTCATCCCGACTTGTCCAAGAC	PPTG_20340StopXhoR	gtcaccctcgagtcaCTTACTCTTTGTGTTCTTTTTGAGCG
PPTG18954	PPTG_18954F	caccatgCCTCATACCGACGAGTCTCAAC	PPTG_18954r	CTTTTTCCCCGTAGCTTTATTTTTAAACG
